# Efficacy of rehabilitation initiated in the early phase after simultaneous deceased donor liver and kidney transplantation: A case report

**DOI:** 10.1097/MD.0000000000035324

**Published:** 2023-09-22

**Authors:** Shinya Tanaka, Yota Mizuno, Shusuke Nojiri, Daiki Futamura, Motoki Nagaya, Yoshihiro Nishida, Yuta Sano, Shohei Ishida, Masashi Kato, Nobuhiko Kurata, Kanta Jobara, Yasuhiro Fujimoto, Yasuhiro Ogura

**Affiliations:** a Department of Rehabilitation, Nagoya University Hospital, Nagoya, Japan; b Department of Orthopaedic Surgery, Nagoya University Graduate School of Medicine, Nagoya, Japan; c Department of Urology, Nagoya University Graduate School of Medicine, Nagoya, Japan; d Department of Transplantation Surgery, Nagoya University Hospital, Nagoya, Japan.

**Keywords:** combined liver and kidney transplantation, exercise, pain, physical performance, rehabilitation

## Abstract

**Rationale:**

The purpose of this case report is to describe a case of successful early rehabilitation intervention for simultaneous liver and kidney transplantation (SLKT).

**Patient concerns:**

A 51-year-old Japanese man was diagnosed with Caroli disease 27 years ago. Hemodialysis was introduced due to end-stage renal disease 17 years ago.

**Diagnoses:**

After successful SLKT, the patient was extubated on postoperative day (POD) 1, liberated from dialysis on POD 4, and discharged from the intensive care unit on POD 9.

**Interventions:**

Supervised rehabilitation was started on POD 2, and the patient was able to walk 100 m on POD 9. Electrical muscle stimulation therapy was started to improve muscle weakness in both legs on POD 16, and aerobic exercise using a cycle-ergometer was started on POD 24.

**Outcomes:**

The 6-minute walking distance improved from 324 m on POD 14 to 501 m on POD 28. The patient could walk 4000 to 5000 steps per day at hospital discharge, and was discharged home on POD 32. There were no adverse events, including worsening hepatic or renal function, during the rehabilitation period. One month after discharge, the patient was able to perform 30 to 40 minutes of aerobic exercise every day, and returned to work 5 months after discharge.

**Lessons:**

This case shows that early rehabilitation intervention immediately after SLKT safely and rapidly improved physical performance without adverse events. The results in the present case suggest that regular physical assessment and appropriate interventions with a variety of exercise modalities can contribute to improved physical performance in SLKT patients.

## 1. Introduction

Simultaneous liver and kidney transplantation (SLKT) is the treatment of choice for patients with end-stage liver and kidney disease, and currently more than 400 SLKT procedures are performed annually in the USA and Europe.^[1,2]^ SLKT is well known to improve prognosis,^[3]^ but both physical performance and physical activity, which are closely related to activities of daily living (ADL) and quality of life, remain disturbed for some time after transplantation.^[4,5]^ Meta-analyses have shown that exercise therapy improves physical performance and health-related quality of life in stable patients after liver or kidney transplantation.^[6,7]^ Although recent studies reported that rehabilitation initiated in the early phase after liver or kidney transplantation is both safe and efficacious,^[8,9]^ the effects of early rehabilitation intervention following SLKT have yet to be elucidated.

This report presents a case of successful early rehabilitation intervention for SLKT.

## 2. Case report

A 51-year-old Japanese man with episodes of epigastric pain and fever was diagnosed with Caroli disease by liver biopsy 27 years ago. Hemodialysis was introduced due to end-stage renal disease and he was placed on a national waiting list for renal transplantation 17 years ago. As he had been hospitalized several times a year over the past 7 years for treatment of cholangitis related to Caroli disease, his transplant waiting status was changed from renal transplant candidate to SLKT candidate because of his medically untreatable cholangitis 4 years ago. He had continued treatment for vasospastic angina and choledocholithiasis. One month prior to SLKT, he experienced a transplant withdrawal due to novel coronavirus disease 2019 infection. Although his Model for End-Stage Liver Disease (MELD) score at transplant and Child-Pugh score were 15 and 6, respectively, standard exception criteria for allocation were applied. ADL was independent and he was working as an office worker.

**Figure 1.**
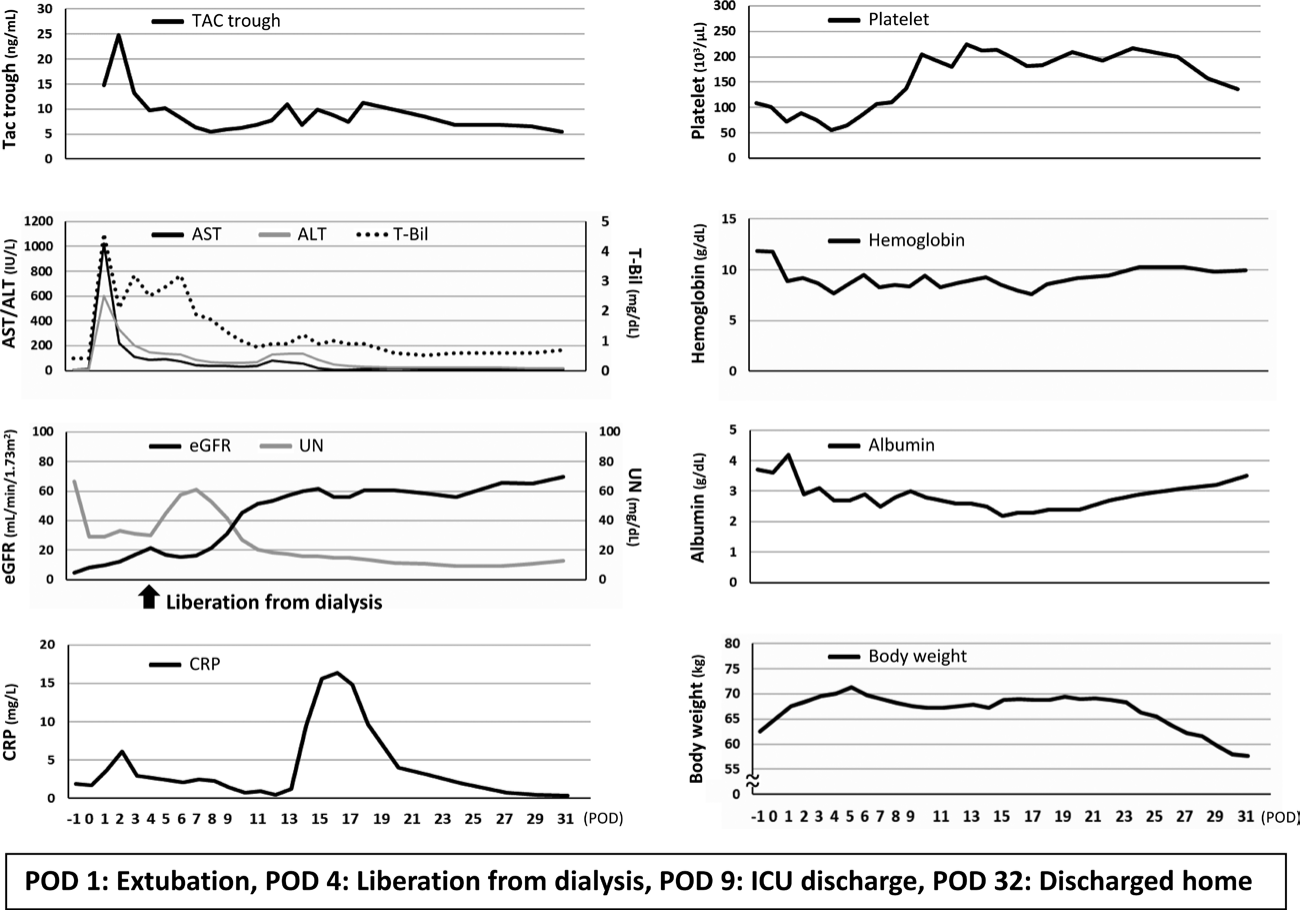
Time course of laboratory data and body weight. ALT = alanine aminotransferase, AST = aspartate aminotransferase, CRP = C-reactive protein, eGFR = estimated glomerular filtration rate, ICU = intensive care unit, POD = postoperative day, TAC = tacrolimus, T-Bil = total bilirubin, UN = urea nitrogen.

In August 2022, the patient underwent successful deceased donor SLKT. The donor was a woman in her 50s who was brain dead due to cerebral hemorrhage. Standard deceased donor liver transplantation was performed with a full-sized liver graft weighing 1078 g (1.66% graft-to-recipient weight ratio), and operation time and blood loss were 522 minutes and 5214 mL, respectively. Subsequently, the graft kidney was transplanted into the right iliac fossa. The postoperative course was uneventful except for infectious complications, which required administration of cefmetazole for the treatment of suspicion of cholangitis on days 12 to 17 (Fig. 1). The patient was extubated on postoperative day (POD) 1, liberated from dialysis on POD 4, discharged from the intensive care unit (ICU) on POD 9, and discharged home on POD 32.

**Figure 2.**
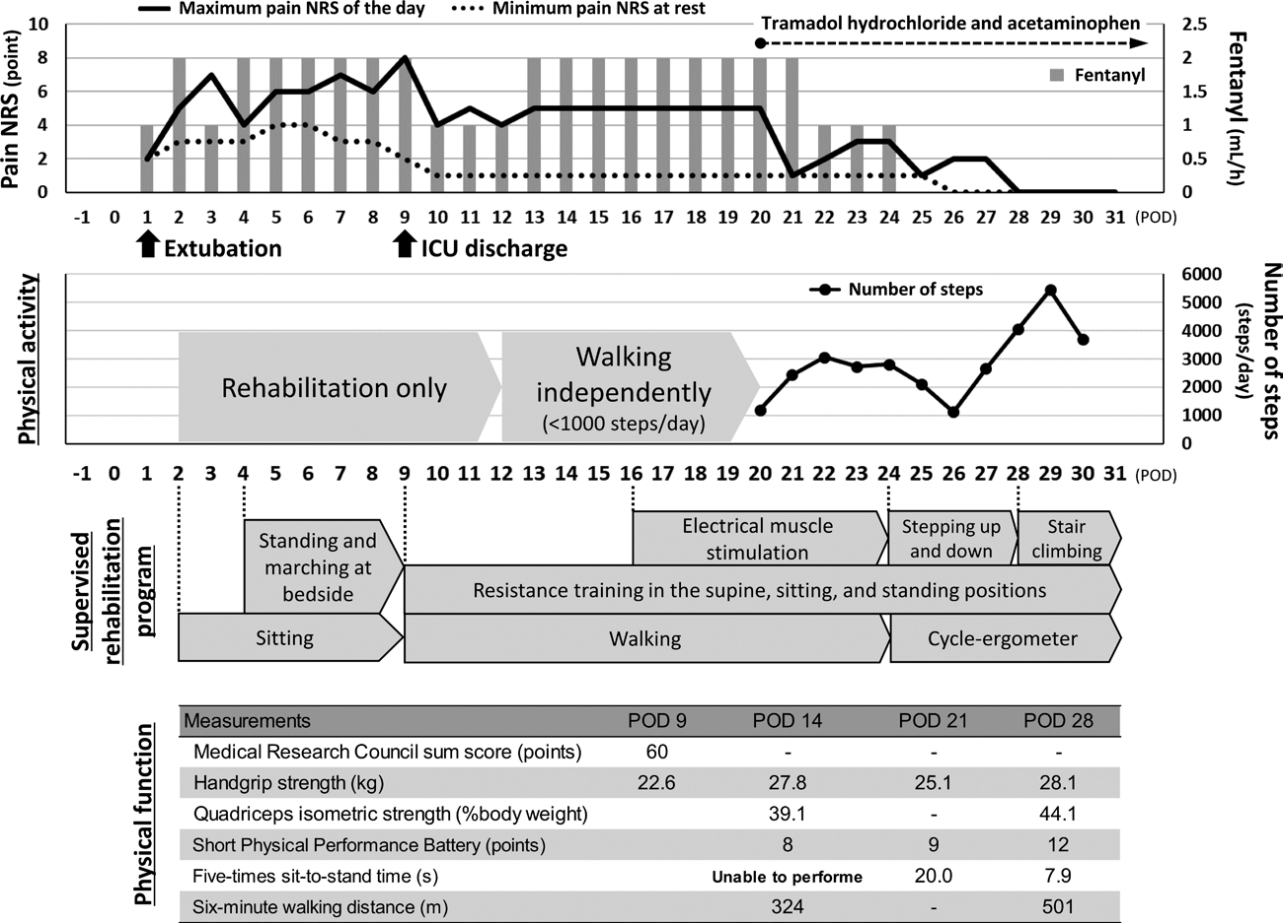
Clinical course of rehabilitation. ICU = intensive care unit, NRS = numerical rating scale, POD = postoperative day.

**Figure 3.**
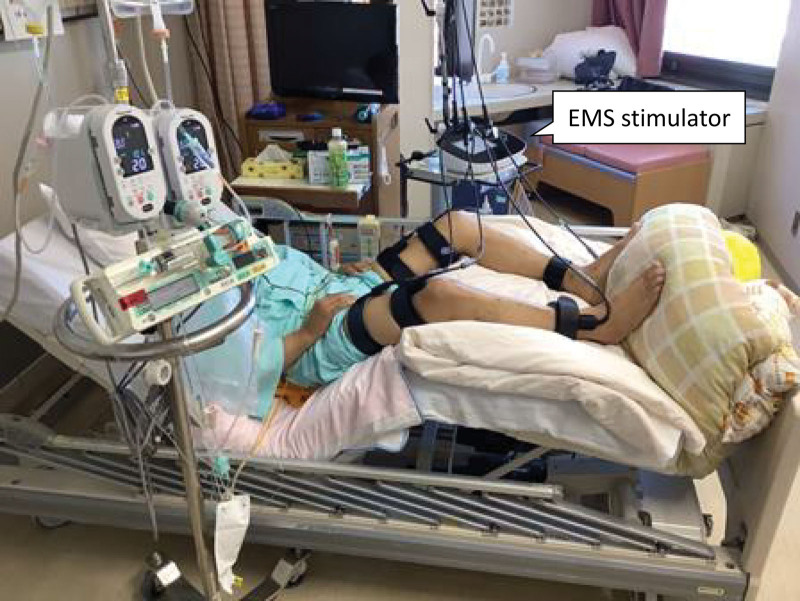
Electrical muscle stimulation for muscles of the lower extremities. Electrical muscle stimulation (EMS) was applied to all muscle groups of both legs including the quadriceps femoris, hamstrings, tibialis anterior, and triceps surae muscles, using a belt electrode skeletal muscle stimulation system (G-TES; Homer Ion Co., Ltd., Tokyo, Japan).

The postoperative course of rehabilitation is shown in Figure 2. Supervised rehabilitation by physical therapists was started on POD 2 at a frequency of 6 days per week, starting with active muscle training in bed and sitting on the edge of the bed. Standing and marching on the spot at the bedside were started on POD 4, and the patient was able to sit on a chair for 60 minutes and perform 200 steps on POD 8. Due to severe pain in the ICU, analgesics were administered before exercise to help the patient tolerate rehabilitation-related activities. The patient started walking on the day of ICU discharge (POD 9), and was able to walk 100 m, which was extended by 100 m each day thereafter. The patient was allowed to walk independently on POD 12, and began measuring physical activity using a triaxial accelerometer. The results of physical function assessment on POD 14 showed reduced lower extremity muscle strength. The following day, the patient was scheduled for a training session to increase lower limb strength, but it was difficult to perform a sufficient amount of exercise due to prolonged pain on exertion. Therefore, electrical muscle stimulation (EMS) therapy was started on POD 16 to improve muscle weakness in both legs (Fig. 3). The output intensity was set to the maximum value without discomfort or pain, and a total of 9 EMS sessions were performed for 30 minutes, 1 to 2 times per day. In addition, resistance training in the supine position for muscles of the upper and lower extremities using elastic bands was added to the voluntary training program. As the patient persistently suffered discomfort and decreased physical activity due to pain, tramadol hydrochloride and acetaminophen were started on POD 20. These analgesics improved the pain on exertion, and the patient began stepping up and down exercise and aerobic exercise using a cycle-ergometer with a workload of 30 W for 10 minutes on POD 24. The exercise intensity was prescribed at a rating of perceived exertion (RPE) of 4 (somewhat hard) on the modified Borg RPE scale (range: 0–10). The patient performed stair climbing exercise and cycle-ergometer exercise (workload of 40 W for 20 minutes) at hospital discharge. On POD 28, physical function assessment showed improvement of lower extremity muscle strength and walking endurance (Fig. 2). The Barthel Index was 100 (independent in basic ADL) and the patient could walk 4000 to 5000 steps per day at hospital discharge. There were no adverse events, including worsening hepatic or renal function, during the rehabilitation period. One month after hospital discharge, the patient was able to perform 30 to 40 minutes of aerobic exercise (walking or cycling) every day, and the EuroQol 5-dimension 5-level score had improved from 0.89 at discharge to 1.00. The patient returned to work 5 months after discharge.

## 3. Discussion

This is the first case report regarding the efficacy and safety of rehabilitation initiated in the early phase after SLKT. SLKT can improve life expectancy,^[3]^ but the US Multicenter SLKT Consortium cohort study reported that early hospitalization was very common after SLKT and discharge to a subacute rehabilitation facility after SLKT was independently associated with early hospitalization.^[10]^ It is recommended that efforts and resources should be focused on identifying SLKT patients at high risk of early hospitalization with optimization of pre-discharge care, discharge planning, and long-term follow-up.^[10]^ Previous studies showed that preoperative MELD score and physical performance were associated with the recovery of physical performance after liver transplantation.^[4,11]^ The patient described here had a low preoperative MELD score and was able to maintain physical performance to work as an office worker, which likely facilitated rehabilitation progress, and as a result, the patient was discharged home.

Previous studies reported that rehabilitation in the acute postoperative period following liver or kidney transplantation appears safe, tolerable, and feasible.^[8,9]^ Acute postoperative pain is common,^[12]^ and enhanced recovery after surgery programs recommend the use of multimodal analgesia not only for postoperative pain control, but also to facilitate mobilization and faster recovery after surgery.^[13,14]^ EMS initiated in hospital during the period of bed rest and inactivity when the majority of physical muscle reduction occurs can be used as an adjunct or bridge to rehabilitation to maintain muscle mass.^[15]^ The results in the present case suggest that regular physical assessment and appropriate interventions with a variety of exercise modalities can contribute to improved physical performance in SLKT patients.

This case report shows that early rehabilitation intervention immediately after SLKT can improve physical performance both safely and rapidly without adverse events. Further studies are needed to validate the effects of early rehabilitation in more vulnerable patients and in larger cohorts.

## Author contributions

**Conceptualization:** Shinya Tanaka, Yota Mizuno.

**Data curation:** Shusuke Nojiri, Daiki Futamura.

**Funding acquisition:** Shinya Tanaka.

**Investigation:** Yuta Sano, Shohei Ishida, Nobuhiko Kurata, Kanta Jobara, Yasuhiro Fujimoto.

**Project administration:** Yoshihiro Nishida, Yasuhiro Ogura.

**Supervision:** Motoki Nagaya, Yasuhiro Ogura.

**Writing – original draft:** Shinya Tanaka.

**Writing – review & editing:** Masashi Kato, Yasuhiro Ogura.
